# GDP Release Preferentially Occurs on the Phosphate Side in Heterotrimeric G-proteins

**DOI:** 10.1371/journal.pcbi.1002595

**Published:** 2012-07-19

**Authors:** Maxime Louet, Jean Martinez, Nicolas Floquet

**Affiliations:** Institut des Biomolécules Max Mousseron (IBMM), CNRS UMR5247, Université Montpellier 1, Université Montpellier 2, Faculté de Pharmacie, Montpellier, France; University of Houston, United States of America

## Abstract

After extra-cellular stimulation of G-Protein Coupled Receptors (GPCRs), GDP/GTP exchange appears as the key, rate limiting step of the intracellular activation cycle of heterotrimeric G-proteins. Despite the availability of a large number of X-ray structures, the mechanism of GDP release out of heterotrimeric G-proteins still remains unknown at the molecular level. Starting from the available X-ray structure, extensive unconstrained/constrained molecular dynamics simulations were performed on the complete membrane-anchored Gi heterotrimer complexed to GDP, for a total simulation time overcoming 500 ns. By combining Targeted Molecular Dynamics (TMD) and free energy profiles reconstruction by umbrella sampling, our data suggest that the release of GDP was much more favored on its phosphate side. Interestingly, upon the forced extraction of GDP on this side, the whole protein encountered large, collective motions in perfect agreement with those we described previously including a domain to domain motion between the two ras-like and helical sub-domains of G_α_.

## Author Summary

Despite the availability of many structural and biochemical data, the activation of G-proteins remains to be understood at the molecular level. We used a computation tool to decipher the first limiting step of this activation: GDP release. Combining different methods of analysis, we propose that the GDP exit occurs on its phosphate side. This study helped to rationalize some experimental observations from the literature and opens many perspectives concerning the study of G-proteins activation and their putative inhibition.

## Introduction

**Figure 1:**
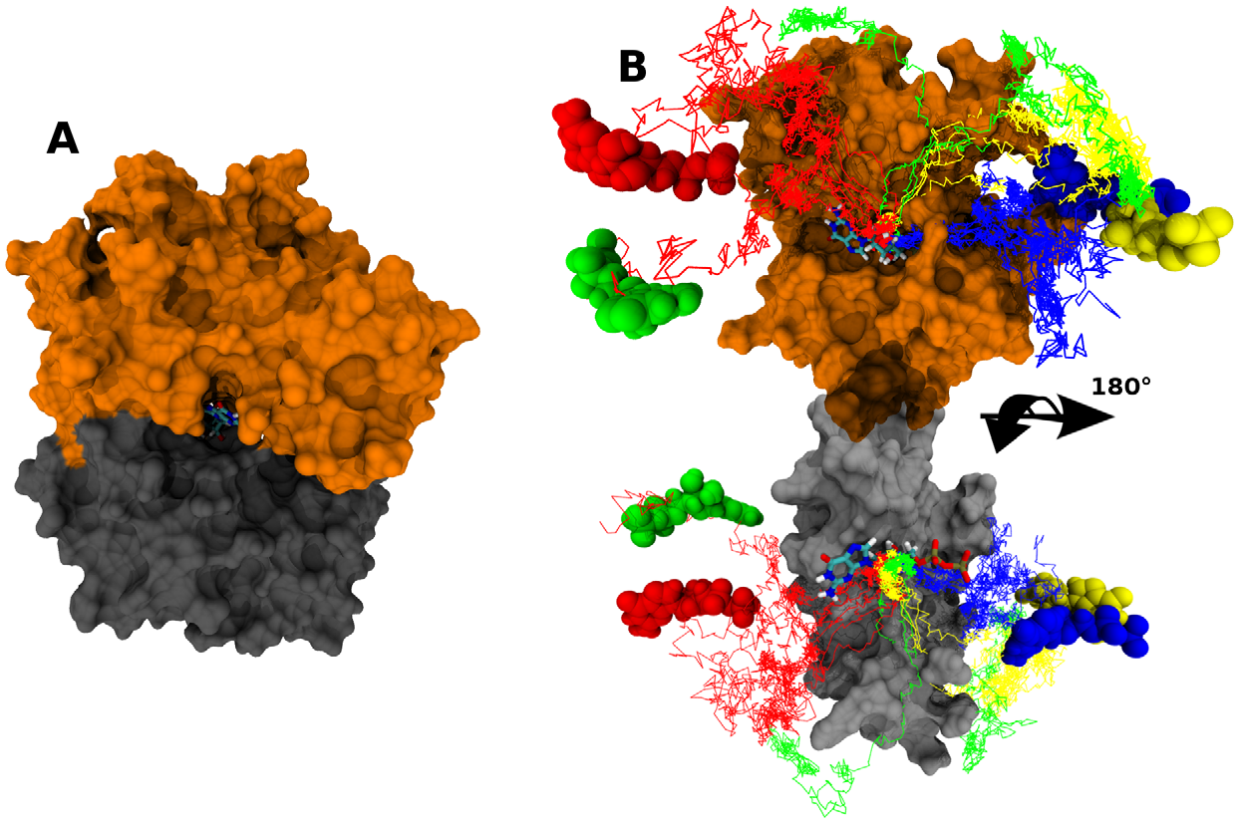
A) Graphical representation of the G_α_ subunit of the Gi heterotrimer. The Ras like and helical domains were reported in orange and black respectively. The GDP, at the interface of the two G_α_ sub-domains was colored according to its atom types. B) Representation of the different positions of the GDP used to extract it out from its binding pocket by the TMD approach (red, blue, yellow, green). To better appreciate the motions of the GDP along each resulting TMD trajectory (colored lines), two different views were reported on the same figure after separation of the two sub-domains of G_α_.

**Figure 2:**
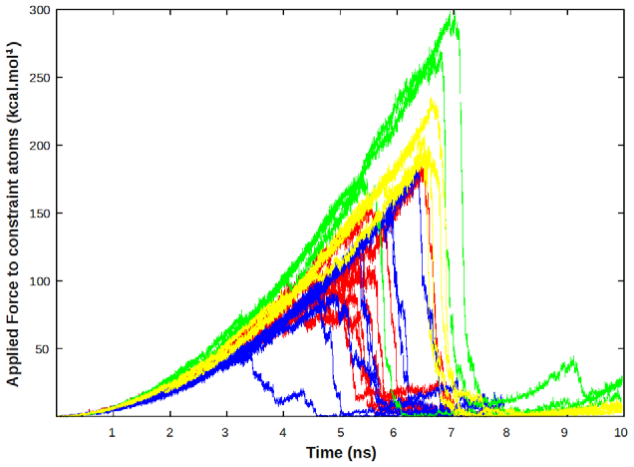
Plot of the forces that were necessary to unbind the GDP out from its initial pocket along each of the TMD trajectories.

In the intracellular compartment, activation of membrane-anchored heterotrimeric G-proteins involves an exchange between GDP and GTP molecules in the G_α_ subunit. This rapid exchange promotes the dissociation of G_α_ from G_βγ_ [1]. GDP release out of G_α_ is “catalyzed” by the direct interaction of the whole heterotrimer with an activated G-protein Coupled Receptor (GPCR) and appears as the rate limiting step [2]. This interaction mainly involves the C-terminal helix of G_α_ as shown by biochemical and structural data [3], [4]. Although many X-ray structures are now available in the Protein Data Bank that describe either G-proteins [5], [6], GPCRs [7], [8], or more recently, their putative interactions [9], the possible mechanism of GDP release still remains unknown at the molecular level. Among other unsolved questions, the exit side of GDP is still debated. Indeed, the GDP ligand lies at the interface between the two ras-like and helical sub-domains of G_α_ (see Figure 1A); addition of hydrogen atoms lacking in the X-ray structure results in a GDP solvent accessible surface of only 2.60 Å^2^ or18.32 Å^2^ on the base or phosphate sides, respectively (representing only ∼4% of the maximum SASA of the GDP, which is 550 Å^2^). A simple visual inspection of the conserved fold of heterotrimeric G-proteins thus suggests two possible exit pathways, either on the base or on the phosphate sides. Recently, the X-Ray structure of the complex between Gs_αβγ_ and the beta-2 adrenergic receptor brought some elements on the GDP-free state of an heterotrimeric G-protein in complex with a GPCR [9]. In this structure, the inter-domain interface was surprisingly completely open, due to a large rigid-body rotation of ∼130° of the helical sub-domain of Gs_α_. As reported recently, this high flexibility appears only in the absence of nucleotide whereas the presence of GDP or GTP favors the stabilization of the α-helical domain on the ras-like domain of Gs_α_ [10]. In this study we were interested in the unbinding process of GDP from the Gi_αβγ_ complex by using extensive molecular dynamics (MD) simulations and reconstruction of free energy profiles along the different putative exit pathways, for a total simulation time overcoming 500 ns (Figure 2). The inactive Gi_αβγ_, GDP bound complex (PDB:1GP2) [5] was equilibrated through a first unconstrained MD trajectory of 40 ns. The ending point of this first simulation was then used to extract the GDP out from its initial position, toward four different directions, by using Targeted Molecular Dynamics (TMD) simulations [11]. It was concluded that this method was successful in generating highly diverse exit pathways for the ligand, as reported in Figure 1B. For each extraction pathway, about 25 intermediate positions of the GDP were further selected and used as starting points for 0.5 ns constrained MD, allowing the reconstruction of free energy profiles using the WHAM algorithm [12]. The sampling of the system was good as proven by a quasi-harmonic analysis of all concatenated data which reproduced the intrinsic, large collective motions we described previously for the same complex [13]. Interestingly, our calculations supported a much easier extraction of the GDP on the phosphate side. Moreover, the forced extraction of GDP on this side promoted large amplitude motions of the protein that were in close agreement with those we described previously as putatively involved in GDP release [13].

## Results

### Description of the equilibrated model after 40 ns MD

The PDB:1GP2 structure was anchored to the membrane as described previously [13] and was subjected to a first all-atoms 40 ns unconstrained Molecular Dynamics (MD) trajectory. At the end of this simulation, we concluded to a significant increase of the GDP total Solvent Accessible Surface Area from 4% to 10% (∼50 Å^2^). This increase was particularly due to some water molecules entering into the binding pocket. This was not surprising as many water molecules were observed in this pocket among available X-ray structures. The GDP position itself was subjected to significant rearrangements. Among these modifications, the ligand lied down into the pocket, but still conserved its main interactions with surrounding residues. In particular, the Phosphate loop (P-loop) was slightly translated, Glu43 establishing a closer interaction with Arg178. In the same time, Asn149 and Asp150 lying at the C-terminus of helix E were also re-oriented, the former inducing a direct interaction with the sugar 3′ hydroxyl of GDP. This was in agreement with the high B-factors observed for the side-chains of these two residues in the PDB:1GP2 X-ray structure. We also observed the formation of an additional helix-turn for helix E, as it can be guessed after a structural alignment of all available G_α_ X-ray structures. Compared to its starting position, Ser47 was reoriented toward the α-phosphate of GDP, so forming an additional interaction that was not seen in the initial X-ray structure. The last significant change concerned the purine base of GDP, which was significantly rotated (∼45°) compared to the starting model, breaking its interaction with Asp272, replaced by water molecules. Quantitatively, the Root Mean Square Deviation (RMSD) computed on GDP atoms between its initial and final positions was 1.8 Å, whereas the RMSD computed on surrounding residues in a sphere of 5 Å was 2.5 Å. This significant change of orientation of the GDP ligand could also have been expected from its high B-factors in many available X-ray structures. Finally, the computed energy of interaction between the GDP and its surrounding residues was more important at the end of the trajectory than in the starting X-ray structure (−873 kcal.mol^−1^ *versus* −424 kcal.mol^−1^) indicating that the observed rearrangements were energetically favorable. Importantly, following of the RMSDs computed either for the protein, the GDP or the GDP binding pocket also argued for a properly equilibrated model at the end of this trajectory.

### Extraction of GDP by targeted molecular dynamics

To extract the GDP ligand out from its binding pocket, we first used Targeted Molecular Dynamics (TMD) simulations [11]. These simulations helped us to generate a large set of intermediate positions of the GDP along different putative exit pathways. Because experimental data were lacking concerning these putative exit pathways of GDP, many possible directions were tested (See Figure 1B). Initially, we expected to perform a clustering analysis on protein atoms to select different possible starting points. However, after equilibration, variations of the RMSD of the protein was only 1 Å all along the trajectory, thus preventing the selection of significantly different starting conformations. Accordingly, the ending conformation of the 40 ns MD was used as a starting point in each case.

The final positions of GDP that were arbitrary chosen to drive the ligand from its bound to unbound states are reported in Figure 1B (blue, red, green and yellow spheres).

In TMD simulations, the movement of the ligand was directly driven by the RMSD difference between its successively observed positions and its final targeted one. Thus, because an identical value of RMSD should correspond to different positions of the ligand, the explored pathways could be significantly different among TMDs, especially when a low force constant of 0.5 kcal.mol^−1^.Å^−2^.atom^−1^ was used as it is the case in the present study. This permitted to generate highly diverse pathways for the GDP. At this step, three groups of pathways were clearly identified. The red group of pathways corresponded to an exit of GDP along its base side. The blue group of pathways corresponded to an exit of GDP along the phosphate side. Yellow and green pathways were together forming a subgroup also directing towards the phosphate side (Figure 1B).

At the beginning of this study, it was expected that variations of the force applied to the ligand might be sufficient to reflect the most significant features along the different explored pathways. Unfortunately, because the ligand was strongly bound to its pocket, this force was observed, in all cases, as continuously increasing in the first stage of each simulation until reaching a quite high value of 300 kcal.mol^−1^, necessary to dislocate the GDP (see Figure 2). This force was particularly high for yellow and green pathways that required more important conformational changes of the protein. This unexpected behavior both led unfortunately to meaningless force profiles and to a discontinuity in the time spend in each successive region along the explored pathways.

In agreement, no detailed analysis was possible on these simulations. Nevertheless, to qualitatively determine the most favorable exit pathways for the GDP, we then used umbrella sampling. A set of ∼25 intermediate positions of the GDP was selected along each TMD trajectory. This was achieved by measuring the distance of GDP to the center of mass of its binding pocket as described in the methods section. Before going further in the study, we carefully verified that these positions were properly distributed along each trajectory.

### GDP preferentially exited on the phosphate side

The Weighted Histogram Analysis Method (WHAM) [12] was used to compute free energy profiles along each of the TMD simulations. In this case, the method was used to describe the easiness/difficulty for the GDP to stay in each of its selected intermediate positions along each of the putative extraction pathways. A WHAM is based on the respect or not of an applied harmonic constraint. In our case, this constraint was a distance between the center of mass of the GDP binding pocket and either the N2 (base side: red pathways in Figure 1B) or P1 (phosphate side: blue, yellow and green pathways in Figure 1B) atoms of the ligand. After different trials, a low constant value of 10 kcal.mol^−1^.Å^−2^ was selected for the applied force.

**Figure 3:**
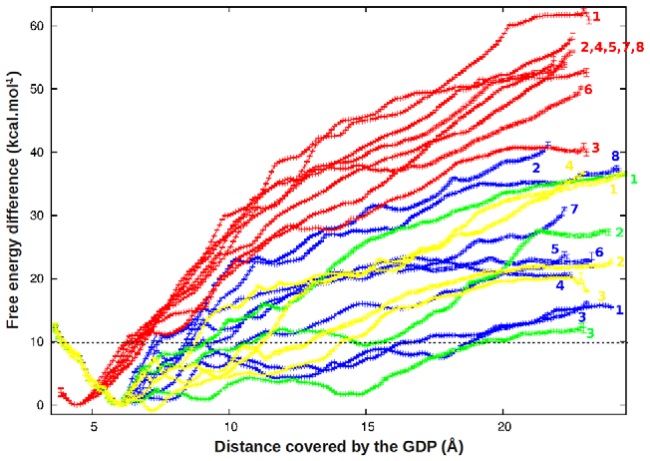
Free energy profiles obtained after analysis of the WHAM. The free energies are given as a function of the distance between the GDP and the center of mass of its initial binding pocket. The dashed line represents the experimental value that was described for GDP unbinding (10 kcal.mol^−1^).

Because of the upper explicated discontinuities along the different extraction pathways, some other intermediate points were added artificially by applying a slightly different constraint to the closest available points. After this step, we verified that two successive constrained positions of the GDP were at a maximal distance difference of 0.5 Å, so recreating a continuity of the data. All the so-built initial conformations were used for constrained molecular dynamics simulations, each lasting 0.5 ns. It was then verified that the chosen points led to a proper covering of the final histograms for the subsequent WHAM (see Figure S1, Figure S2 and Figure S3). The free energy profiles resulting from the WHAM were reported in Figure 3. As a first point, and in agreement with our observations described previously, it was concluded that the obtained conformation at 40 ns was located in a potential depth as compared to the X-ray crystallographic structure. More significantly, the results clearly indicated a preferred exit on the phosphate side (blue and yellow+green profiles) whereas an exit on the base side was qualitatively more difficult (red profiles). Interestingly, the three best profiles (blue 1+3 and green 3) corresponded to the phosphate side and to a predicted free energy cost of about +15 kcal.mol^−1^ in good agreement with the dissociation constant of 0.2 µM measured on the Gi protein at ambient temperature (∼10 kcal.mol^−1^) [14].

**Figure 4:**
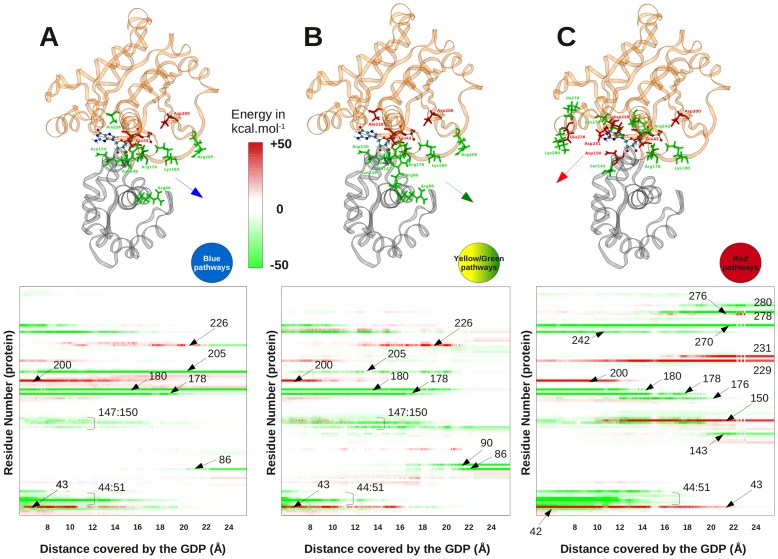
Non-bonded energies of interaction between the GDP and surrounding G_α_ residues along representative groups of pathways (A) blue group of pathways, (B) yellow+green groups of pathways and (C) red group of pathways. On the top of the figure, key residues of G_α_ were colored in red or green according to their positive or negative non-bonded interactions with the ligand. On the bottom, the interaction energies between the ligand and each surrounding residues were plotted as a function of the distance covered by the GDP, every 0.02 Ångström.

To better visualize the interactions established between the GDP and its environment, a list of residues was further built that included all the protein residues lying at a maximum distance of 4 Å of the GDP, as observed along all the explored exit pathways. Non-bonded (Van Der Waals and electrostatic) energies were then re-computed for each GDP:protein residue pair with NAMD [15]. Strong negative energy of interaction was reported in green, whereas a positive energy (including charge:charge repulsions) was reported in red. Results of this analysis can be seen in Figure 4. For more clarity, only three representative interaction energy profiles were reported in this figure that corresponded to the best profiles obtained for each of the blue, green+yellow, or red groups of pathways, respectively. Importantly, it was verified that these profiles of interactions were well representative of all the other computed energy patterns from other trajectories, with correlation coefficients of ∼0.9+−0.05 (blue pathways), ∼0.9+−0.05 (red pathways) and ∼0.75+−0.14 (green+yellow pathways). These high correlation values nicely confirmed that the chosen exit pathways for GDP corresponded to highly conserved interactions, even if the final free energy profiles shared some discrepancies. Subsequent analyses of these profiles further allowed to extract the most significant events leading to the GDP exit out from its initial binding pocket along these three groups of pathways.

In agreement with the corresponding free energy profiles, an increased number of strong “red”, non favorable interactions was observed on the base side (red group of pathways, Figure 4C). The exit of GDP was first characterized by the loss of some favorable interactions with residues Gly42, Ser44, Gly45, Lys46, Ser47 and Lys51, at a distance of ∼15 Å from the center of mass. These residues all belong to the helix 1 or P-loop of G_α_. In this protein region, a strong repulsion between Glu43 and the Phosphate moiety of GDP was also noticed. Strong interactions were also established with Asn149 and Ser151, involving successively the sugar and then the phosphate moieties of GDP. The change from “good” to “bad” interaction involving Asp150 corresponded to a shift of its interaction with the base to the phosphate moiety of GDP. Interactions with Arg178 and Lys180 were maintained a long time, especially for Arg178 which interacted successively with the α- and then with the β-phosphate of the ligand. At the end of the trajectories, another “good” interaction was created with Arg176. Nevertheless, three additional non favorable interactions were evidenced, first with Asp200 in the first stage of the trajectories that reflected a repulsion with the phosphates, and also with the two Asp229 and Asp231 residues at the end of the simulations. Ser143, Arg242, Lys270, Ile278 and Lys280 played a more favorable role, all along the trajectories. Finally, it was observed that the Glu276 was also a main contributor to the “bad” free energy profiles observed on the base side.

On the phosphate side, including blue and green+yellow pathways, interactions were quite conserved as depicted in Figure 4. Indeed, the mean correlation computed between blue and green+yellow profiles of interactions was 0.58+−0.2 whereas the same computed between blue and red or red and green+yellow was 0.47+−0.05 or 0.47+−0.1, respectively.

On the phosphate side, strong repulsions were only observed with Glu43 and Asp200, at the beginning of simulations. Another repulsion was noted for Ala226 which turned to a “good” interaction at the end of blue profiles. No other strong repulsion was noted on the exit route of GDP. As observed on the base side, strong favorable interactions with Gly42, Ser44, Gly45, Lys46, Ser47 and Lys51 were quickly lost. Repulsion with Glu43 was less significant and even converted into a favorable interaction in the middle of the simulations when the sugar and the base moieties came in close contact. The most favorable interactions included residues Arg86, Arg178, Lys180 and Arg205 driving the exit of the ligand. Other significant contributions were noticed for residues 147 to 150 located at the N-terminus of Helix αE. The role of the two Glu43 and Arg178 residues was particularly interesting as they were strongly interacting during the entire trajectory of 40-ns MD. We observed that extraction of GDP along the phosphate side irremediably led to the breaking of this interaction, thus promoting the separation of the Switch I from the P-loop segment. This separation that could have been expected from a direct visualization of available X-ray data, was not observed during the extraction of GDP along the base side.

Interestingly, some site-directed mutagenesis studies have already shown the importance of some of the upper mentioned residues in GDP release. Among them, an R178M mutant was shown to increase GDP release by 10-fold [16]. S43N mutation in Gt_α_ (Ser47) also increased GDP release [17]. Other candidates could be easily proposed from our calculations, including residues that are not in contact with GDP in the known X-ray structures, but located on its putative exit route.

### GDP-induced motions of the whole heterotrimer

Using normal modes calculations *in vacuo* performed on the whole heterotrimeric protein, we previously proposed that the unbinding of GDP might require (or promote) large, collective motions of the protein [13]. The involved mode (mode 17) described an inter-domain motion intrinsic to the G_α_ subunit, leading to the partial opening of the GDP binding pocket. To confirm or not the importance of such a motion in the release of GDP, we performed Essential Dynamics Analyses (EDA) on our data from TMD simulations. Such type of analysis is usually used to capture the large, collective motions of the system from a single or concatenated MD trajectory [18].

Four ensembles of trajectories were first built by concatenation of the data, namely TMDB (TMD data, blue group of pathways, ∼60 ns), TMDYG (TMD data, yellow+green groups of pathways, ∼70 ns), and TMDR (TMD data, red group of pathways, ∼60 ns). After a Principal Component Analysis (PCA) of the backbone coordinates, only the fifty lowest frequency quasi-modes were retained for further analysis. All the motions described by the obtained quasi-modes were then individually compared to the 20 lowest frequencies “true” normal modes we described previously for the 1GP2 crystallographic structure [13], including the expected mode 17. Importantly, the same forces field was used in both studies. These comparisons were performed through the computation of displacement matrices and correlation coefficients as previously described [13], [19], [20]. We remember here that using this criterion, a correlation of 0.6 corresponds to two highly closely related motions in the cartesian space.

**Figure 5:**
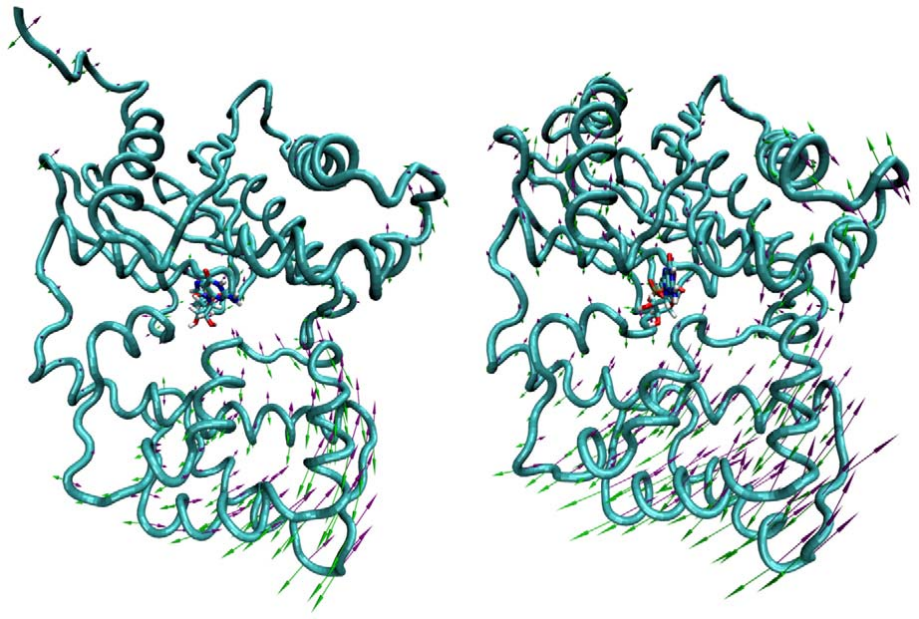
Left: Motions described by the Quasi-Mode 11 when the GDP exited on the phosphate side (blue group of pathways). These motions were highly related to those we described previously by NMA (on the right: mode 17) as putatively involved in GDP release [13].

First, it was concluded that pulling the GDP on the base side (TMDR) led to no significant correlation coefficient (<0.5) between the deduced quasi-modes and any of the previously described NMs. Similar results were concluded after analysis of the TMDYG data. On the contrary, the mode 17 was retrieved with a high correlation coefficient of 0.7 when using the data from TMDB (Quasi-mode number 11) thus confirming its putative role in the Gi heterotrimer activation and GDP release [13]. This motion depicted in Figure 5 corresponded to a concerted motion involving especially the C-terminus, the α4 and the αG helices, as well as the whole helical domain of G_α_. Interestingly, these intrinsic motions also described a separation of the two ras-like and helical sub-domains of G_α_, as strongly suggested by recent experimental observations [10], [21]. Importantly, this motion was not retrieved when performing an EDA of the initial unconstrained MD trajectory of 40 ns, so strongly suggesting that it was induced by the exit of GDP from its initial position.

## Discussion

Obviously G-proteins X-ray structures are available for a long time, the fine mechanism of GDP release at the molecular level still remains unknown. Because it constitutes the rate limiting step for G-proteins activation, it is of crucial interest. In this study, we argue for a GDP release on the phosphate side of the ligand. Key residues involved in this release have been identified, some of them having been already delineated by previously published experimental studies. Other mutants, able to either promote or block the GDP release, should easily be proposed based on our computational results, including Arg205 and other residues located on the N-terminus region of Helix αE. To extend these analyses, closely related calculations on other G-proteins should be performed, notably to explain discrepancies in their GDP release rates. Our results also argue for a steric effect of the Go-Loco peptide of RGS14 (PDB:1KJY), which inhibits GDP release through its binding to the G_α_ subunit [22]. In precedent studies, it was rather suggested that this peptide might block the GDP release by affecting the inter-domain dynamics of G_α_. Interestingly, and despite the fact that it is located on the putative phosphate exit route, the positioning of the Go-Loco peptide was completed by a reorientation of the Switch I region, an outward movement of the Lys180 residue, and by a direct interaction with Asn149, two residues we pointed out as very significant for the GDP release. The importance of Switch I and of its conformational change during the G-protein activation cycle shown is this study was also previously suggested by experiments [23].

Here, we were especially interested by GDP release. Another question logically raised concerning the GTP binding. The accomplishment of such a study related to GTP binding to the G-protein heterotrimer would be more problematic because of the choice of the starting structure. Indeed, as demonstrated recently, the inter-domain dynamics of G_α_ is more significant in the absence of the nucleotide [10], [21]. In agreement, some parts of the X-ray structure that describes an unbound G_α_ subunit (PDB:3SN6) would require to be rebuilt, because of some putative crystallization artifacts [9].

It would also be of interest to understand the exact role of the GDP in the stabilization of the G_α_(ras):G_α_(helical) sub-domains interactions. This study showed how the GDP release on its phosphate side could induce large, collective motions of the whole heterotrimer as shown by Essential Dynamics analyses. Furthermore, this motion was supported by our previous findings [13] and guessed to be involved in G-protein heterotrimeric activation. These motions are also thought to be promoted by the molecular interactions between the G-protein itself and the GPCR, in part through the α4 helix of the G_α_ sub-unit and the ICL3 region of the receptor. Some calculations are actually under progress to understand how the activation of GPCRs could promote G-proteins conformational changes and the subsequent GDP release.

## Materials and Methods

### MD simulation

The 40-ns unconstrained molecular dynamics (MD) simulation was performed on the whole Gi heterotrimer starting with the PDB:1GP2 crystallographic structure [5]. The protein was first inserted into a membrane model composed of 406 1-palmitoyl-2-oleyl-phosphatidyl-choline (POPC) lipids. This insertion was performed through the construction of three lipid-modified anchoring residues on G_α_-Gly2 (myristoyl), G_α_-Cys3 (palmitoyl), and G_γ_-Cys68 (geranylgeranyl). Anchoring residues were preliminary built with the Antechamber and the General Amber Forces field [24], [25]. For the rest of the system, the CHARMM forces field was used including CMAP corrections [26]. The system was solvated in each z-direction with TIP3P water molecules [27]. 17 sodium ions were finally added to fully neutralize the system, allowing the use of the particle mesh Ewald method [28] for the computation of electrostatic interactions. A switching function was applied to Van Der Waals interactions in the range 10–12 Å. The NPT ensemble was used (1.013 bars and 298 K) with Langevin dynamics and a Nosé–Hoover–Langevin piston pressure control. The integration step was set to 1 fs. Interactive molecular dynamics, using NAMD [15] and VMD [29], was used to slowly insert the anchoring residues into the membrane. After a minimization through 5.000 steps of conjugate gradient, the whole system was then equilibrated during a first stage of 2ns MD simulation during which the protein atoms were kept fixed. In a second stage, all constraints were removed, and the simulation was pursued until reaching 40 ns.

### TMD simulations

In TMD simulations [11], the force applied to each of the N atoms of the pulled ligand is of the form:
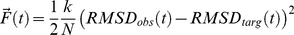
(1)where *k* is a force constant given in kcal.mol^−1^.Å^−2^ and *RMSD_obs_(t)* and *RMSD_targ_(t)* the observed and targeted values of RMSD at a given time step *t*, respectively. RMSDs were computed on the entire set of the *N* ligand atoms. TMD simulations were performed starting from the ending conformation of the 40 ns MD simulation. The used parameters were strictly identical, except for the random seed number generator that was changed to generate different initial velocities. The final targeted positions of the ligand were built by translating and/or rotating the GDP out of its binding pocket, in arbitrary chosen positions, either on the phosphate or on the base sides (see Figure 1B). To avoid translation of the whole protein during TMD simulations, the C_α_ of the four Glu43, Gly45, Thr48 and Arg178 residues of G_α_ were also subjected to the TMD force. Except these residues and GDP, all the remaining atoms of the system were kept free of any constraint. It was concluded that behaviors of residues with constrained C_α_ were not disturbed as their Root Mean Square Fluctuations (RMSFs) were similar to those observed for the 40 ns MD simulation. The applied force constant k was set to 20 kcal.mol^−1^.Å^−2^ (0.5 kcal.mol^−1^.Å^−2^ per atom), a low value in the range of that described in other recent published TMD studies [30], [31].

Each TMD simulation lasted for about 8 to 10 ns, corresponding to a linear decrease in the targeted RMSD of ∼2.5.10^−3^ Å.ps^−1^. It was checked that the GDP ligand was out of the protein at the end of each TMD simulation.

### WHAM

TMD simulations were used to generate intermediate positions of the GDP along different possible exit pathways. These positions were further used to perform umbrella sampling simulations and Weighted Histogram Analyses [12] of the obtained data. The harmonic constraint necessary for the umbrella sampling simulations was defined as a distance between the center of mass of the GDP binding pocket, and either the N2 or P1 atoms of GDP (base or phosphate side). The center of mass (COM) of the GDP pocket was computed from the C_α_ coordinates of residues lying at a maximal distance of 4 Å to the GDP in the initial X-ray crystallographic structure. The harmonic potential used to constrain the GDP at successive distances *d_eq_* from the COM was reported in Eq. 2:
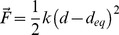
(2)where the force constant *k* was set to 10 kcal.mol^−1^.Å^−2^. Along each TMD, intermediate positions were extracted assuming a minimal increment of 0.5 Å for two successive GDP:COM distances. However, because of the TMD method, this criterion was not always sufficient to get a good coverage of the whole trajectory. To supplement these data, we performed additional simulations by starting from the closest position of GDP and setting up the distance constraint to an intermediate value. To ensure that the GDP fully explored the desired intermediary positions, the force constant *k* was increased to 20 kcal.mol^−1^.Å^−2^. All the obtained starting points were further subjected to a constrained MD simulation of 0.5 ns, once more using the same simulation parameters than for the 40 ns or TMD simulations.

For analyses of the obtained data, the WHAM algorithm [12] was used as implemented in the code distributed by Grossfield (http://membrane.urmc.rochester.edu/wham/).

### Essential Dynamics and comparison of motions

Quasi-modes were derived from the molecular dynamics simulations after computation of the covariance matrices of the mass weighted Cartesian coordinates. In each performed analysis, only the backbone atoms were considered and a total of 50 quasi-modes were extracted with CHARMM. Quasi-modes were then compared to the “true” all-atoms normal modes we described previously [13] and computed with the same forces field as that used in the present study. The direct comparisons of motions described by either modes or quasi-modes were performed by using displacement matrices as previously explained [13]. Briefly, the method consisted to compute 2D matrices that reflected the relative displacements of all pairs of C_α_ atoms between two structures. For each considered mode, these matrices were computed from the two structures obtained at a displacement amplitude of +1 Å in each direction. Correlation coefficients between two matrices were then computed with the Mantel test [32]. With this method, it was assumed that two 2D maps sharing a correlation coefficient greater than 0.5 described highly related motions in the Cartesian Space.

## Supporting Information

Figures S1 Plot of the distances distributions between the GDP and the center of mass of its pocket obtained by umbrella sampling and used for the WHAM (red pathways).
(TIFF)

Figures S2 Plot of the distances distributions between the GDP and the center of mass of its pocket obtained by umbrella sampling and used for the WHAM (green+yellow pathways).
(TIFF)

Figures S3 Plot of the distances distributions between the GDP and the center of mass of its pocket obtained by umbrella sampling and used for the WHAM (blue pathways).
(TIFF)
