# Supplementary figures and images for: GDP Release Preferentially Occurs on the Phosphate Side in Heterotrimeric G-proteins
Source: PLoS Comput Biol. 2012 Jul 19;8(7):e1002595. doi: 10.1371/journal.pcbi.1002595 (PMC3400569; doi:10.1371/journal.pcbi.1002595)

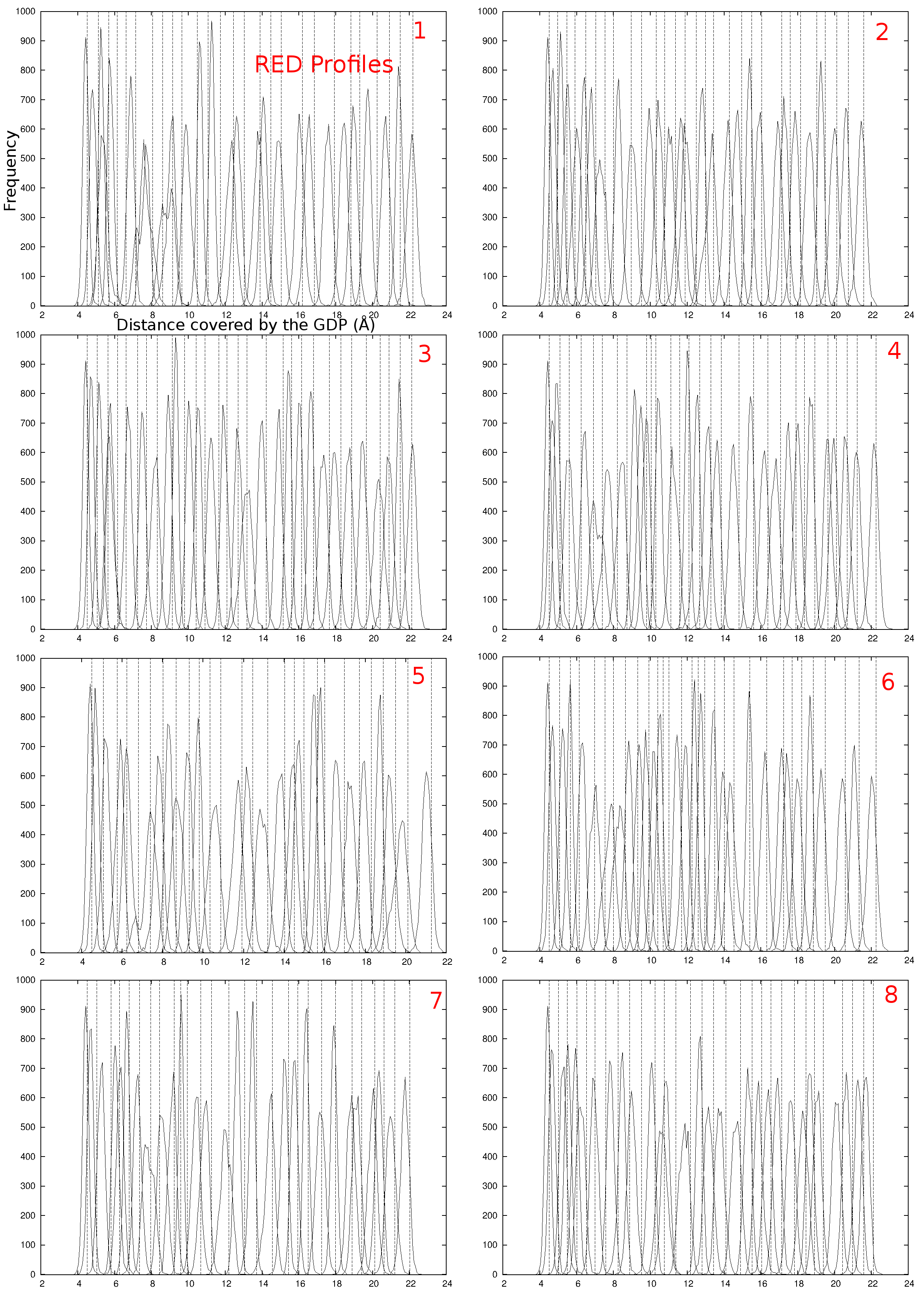

Supplement: Figures S1 — Plot of the distances distributions between the GDP and the center of mass of its pocket obtained by umbrella sampling and used for the WHAM (red pathways). (TIFF) [file pcbi.1002595.s001.tif]

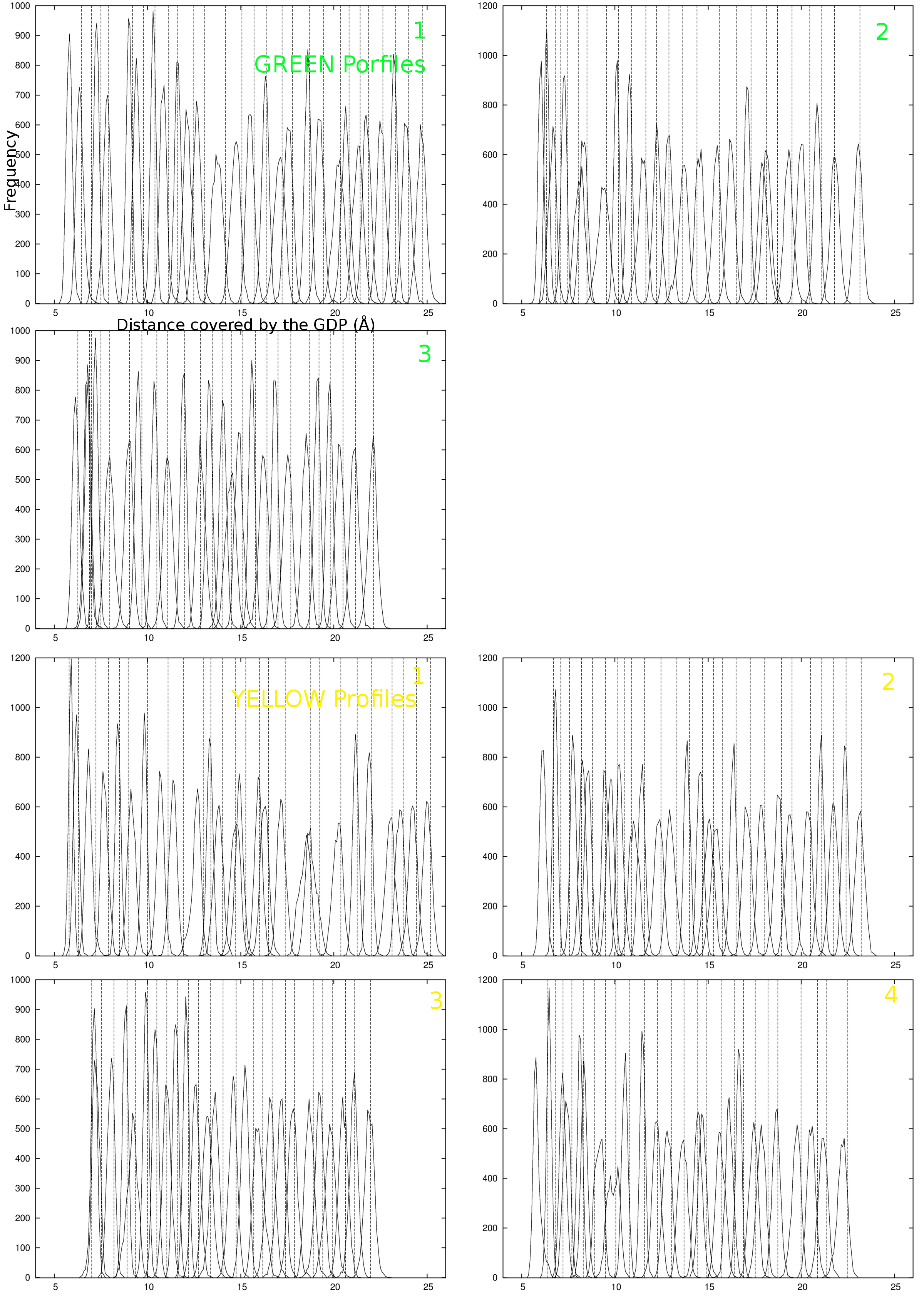

Supplement: Figures S2 — Plot of the distances distributions between the GDP and the center of mass of its pocket obtained by umbrella sampling and used for the WHAM (green+yellow pathways). (TIFF) [file pcbi.1002595.s002.tif]

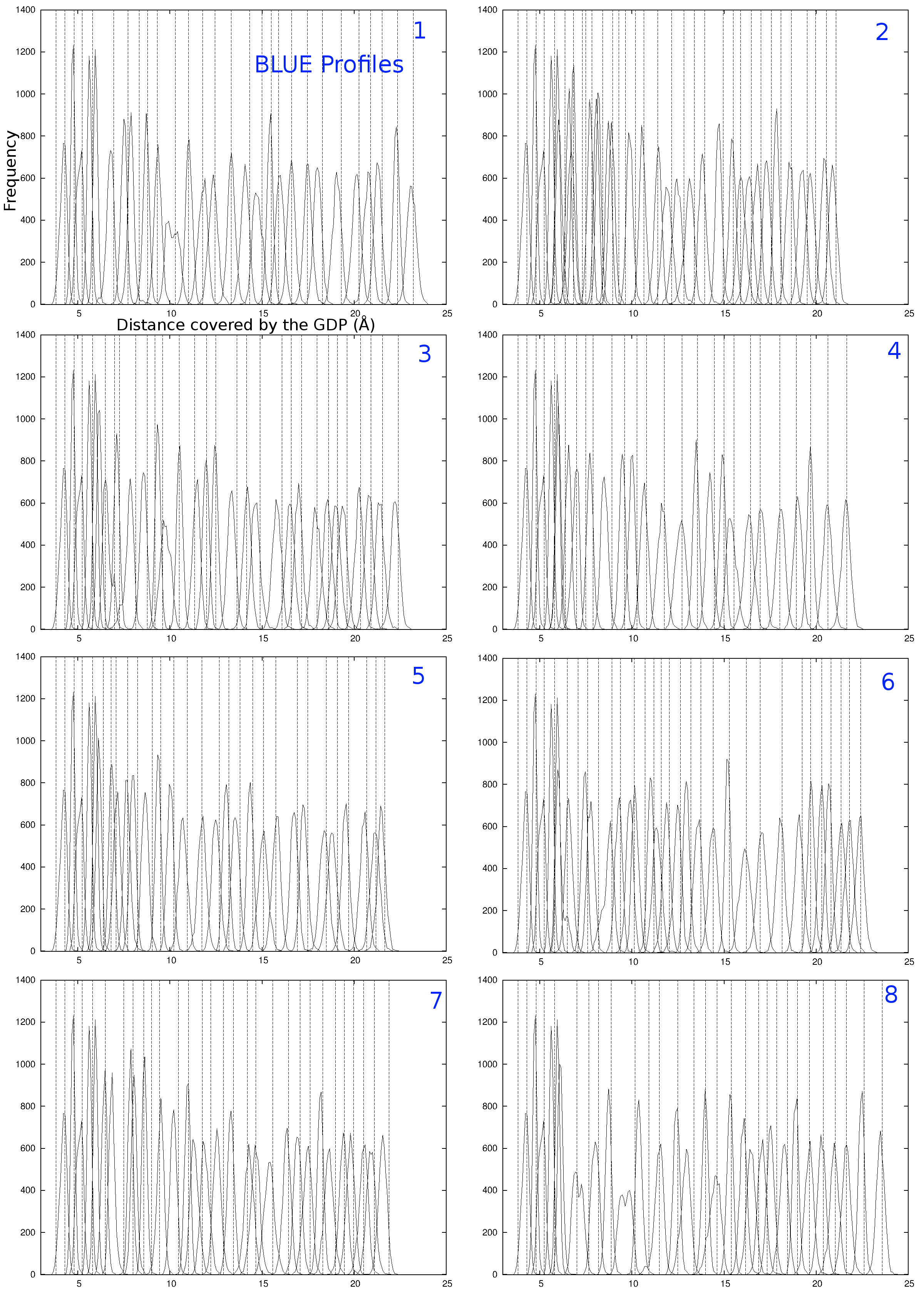

Supplement: Figures S3 — Plot of the distances distributions between the GDP and the center of mass of its pocket obtained by umbrella sampling and used for the WHAM (blue pathways). (TIFF) [file pcbi.1002595.s003.tif]
